# *N*-(4-Chloro­phen­yl)morpholine-4-carboxamide

**DOI:** 10.1107/S1600536811034611

**Published:** 2011-08-27

**Authors:** Yu-Feng Li

**Affiliations:** a Microscale Science Institute, Department of Chemistry and Chemical Engineering, Weifang University, Weifang 261061, People’s Republic of China

## Abstract

In the title mol­ecule, C_11_H_13_ClN_2_O_2_, the morpholine ring has a chair conformation. In the crystal, mol­ecules are linked into chains along [100] by N—H⋯O hydrogen bonds.

## Related literature

For the applications of morpholine compounds, see: Arrieta *et al.* (2007 ▶). For related structures, see: Li (2011*a* ▶,*b* ▶).
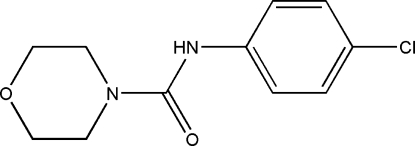


## Experimental

### 

#### Crystal data

C_11_H_13_ClN_2_O_2_
*M* *_r_* = 240.68
Orthorhombic, 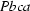

*a* = 9.3359 (19) Å
*b* = 11.105 (2) Å
*c* = 22.426 (5) Å
*V* = 2325.0 (8) Å^3^
*Z* = 8
Mo *K*α radiation
μ = 0.32 mm^−1^
*T* = 293 K
0.26 × 0.19 × 0.18 mm

#### Data collection

Bruker SMART CCD diffractometer
20865 measured reflections
2660 independent reflections
2081 reflections with *I* > 2σ(*I*)
*R*_int_ = 0.059

#### Refinement

*R*[*F* ^2^ > 2σ(*F* ^2^)] = 0.044
*wR*(*F* ^2^) = 0.124
*S* = 1.07
2660 reflections
149 parameters
H atoms treated by a mixture of independent and constrained refinement
Δρ_max_ = 0.40 e Å^−3^
Δρ_min_ = −0.29 e Å^−3^

### 

Data collection: *SMART* (Bruker, 1997 ▶); cell refinement: *SAINT* (Bruker, 1997 ▶); data reduction: *SAINT*; program(s) used to solve structure: *SHELXS97* (Sheldrick, 2008 ▶); program(s) used to refine structure: *SHELXL97* (Sheldrick, 2008 ▶); molecular graphics: *SHELXTL* (Sheldrick, 2008 ▶); software used to prepare material for publication: *SHELXTL*.

## Supplementary Material

Crystal structure: contains datablock(s) global, I. DOI: 10.1107/S1600536811034611/lh5309sup1.cif

Structure factors: contains datablock(s) I. DOI: 10.1107/S1600536811034611/lh5309Isup2.hkl

Supplementary material file. DOI: 10.1107/S1600536811034611/lh5309Isup3.cml

Additional supplementary materials:  crystallographic information; 3D view; checkCIF report

## Figures and Tables

**Table 1:** Hydrogen-bond geometry (Å, °)

*D*—H⋯*A*	*D*—H	H⋯*A*	*D*⋯*A*	*D*—H⋯*A*
N2—H1*N*⋯O2^i^	0.838 (19)	2.114 (19)	2.9226 (19)	162.2 (19)

Symmetry code: (i) 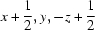.

**Fig. 1.**
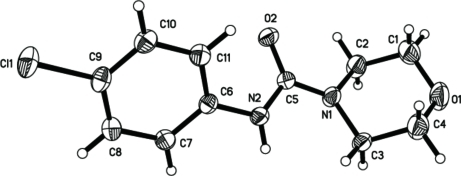
The molecular structure of the title compound showing 30% probability displacement ellipsoids.

## supplementary crystallographic information

### Comment

Morpholine compounds are an important intermediate reagent in organic synthesis (Arrieta *et al.*, 2007). As part of our search for new carboxamide compounds (Li, 2011*a*,*b*) we have determined the crystal structure of the title compound containing a morpholine ring. The molecular structure of the title compound is shown in Fig. 1. The morpholine ring (N1/C1/C2/C3/C4/O1) is in a chair conformation. In the crystal, molecules are linked into chains along [100] by  N—H···O hydrogen bonds.

### Experimental

A mixture of morpholine (0.1 mol), and (4-chlorophenyl)carbamic chloride (0.1 mol) was stirred in refluxing ethanol (20 ml) for 4 h to afford the title compound (0.065 mol, yield 65%). Colourless blocks of the title compound were obtained by recrystallization from ethanol at room temperature.

### Refinement

H atoms were fixed geometrically and allowed to ride on their attached atoms, with C—H distances = 0.93–0.97 Å and with *U*_iso_(H) = 1.2*U*_eq_(C). The H atom bonded to N2 was refined independently with an isotropic displacement parameter.

### Crystal data

**Table:**

C_11_H_13_ClN_2_O_2_	*F*(000) = 1008
*M**_r_* = 240.68	*D*_x_ = 1.375 Mg m^−^^3^
Orthorhombic, *P**b**c**a*	Mo *K*α radiation, λ = 0.71073 Å
Hall symbol: -P 2ac 2ab	Cell parameters from 2081 reflections
*a* = 9.3359 (19) Å	θ = 2.6–27.4°
*b* = 11.105 (2) Å	µ = 0.32 mm^−^^1^
*c* = 22.426 (5) Å	*T* = 293 K
*V* = 2325.0 (8) Å^3^	Block, colorless
*Z* = 8	0.26 × 0.19 × 0.18 mm

### Data collection

**Table:**

Bruker SMART CCD diffractometer	2081 reflections with *I* > 2σ(*I*)
Radiation source: fine-focus sealed tube	*R*_int_ = 0.059
graphite	θ_max_ = 27.5°, θ_min_ = 3.0°
φ and ω scans	*h* = −11→12
20865 measured reflections	*k* = −14→14
2660 independent reflections	*l* = −29→29

### Refinement

**Table:**

Refinement on *F*^2^	Primary atom site location: structure-invariant direct methods
Least-squares matrix: full	Secondary atom site location: difference Fourier map
*R*[*F*^2^ > 2σ(*F*^2^)] = 0.044	Hydrogen site location: inferred from neighbouring sites
*wR*(*F*^2^) = 0.124	H atoms treated by a mixture of independent and constrained refinement
*S* = 1.07	*w* = 1/[σ^2^(*F*_o_^2^) + (0.0583*P*)^2^ + 0.5192*P*] where *P* = (*F*_o_^2^ + 2*F*_c_^2^)/3
2660 reflections	(Δ/σ)_max_ < 0.001
149 parameters	Δρ_max_ = 0.40 e Å^−^^3^
0 restraints	Δρ_min_ = −0.29 e Å^−^^3^

### Special details

**Table:**

Geometry. All e.s.d.'s (except the e.s.d. in the dihedral angle between two l.s. planes) are estimated using the full covariance matrix. The cell e.s.d.'s are taken into account individually in the estimation of e.s.d.'s in distances, angles and torsion angles; correlations between e.s.d.'s in cell parameters are only used when they are defined by crystal symmetry. An approximate (isotropic) treatment of cell e.s.d.'s is used for estimating e.s.d.'s involving l.s. planes.
Refinement. Refinement of *F*^2^ against ALL reflections. The weighted *R*-factor *wR* and goodness of fit *S* are based on *F*^2^, conventional *R*-factors *R* are based on *F*, with *F* set to zero for negative *F*^2^. The threshold expression of *F*^2^ > σ(*F*^2^) is used only for calculating *R*-factors(gt) *etc*. and is not relevant to the choice of reflections for refinement. *R*-factors based on *F*^2^ are statistically about twice as large as those based on *F*, and *R*- factors based on ALL data will be even larger.

### Fractional atomic coordinates and isotropic or equivalent isotropic displacement parameters (Å^2^)

**Table:**

	*x*	*y*	*z*	*U*_iso_*/*U*_eq_	
Cl1	0.12457 (6)	0.60377 (5)	0.44436 (2)	0.0656 (2)	
O1	0.41840 (19)	0.16232 (19)	0.04233 (6)	0.0854 (6)	
O2	0.14298 (12)	0.24548 (12)	0.21323 (5)	0.0505 (3)	
N1	0.34301 (15)	0.19345 (14)	0.16222 (6)	0.0473 (4)	
N2	0.35998 (15)	0.31812 (13)	0.24422 (6)	0.0407 (3)	
C1	0.2740 (3)	0.1513 (3)	0.06064 (9)	0.0782 (7)	
H1A	0.2252	0.0940	0.0351	0.094*	
H1B	0.2264	0.2285	0.0567	0.094*	
C2	0.2651 (2)	0.1097 (2)	0.12415 (8)	0.0598 (5)	
H2B	0.1657	0.1058	0.1365	0.072*	
H2C	0.3061	0.0298	0.1277	0.072*	
C3	0.48960 (18)	0.21365 (17)	0.14282 (7)	0.0477 (4)	
H3A	0.5456	0.1411	0.1489	0.057*	
H3B	0.5322	0.2777	0.1663	0.057*	
C4	0.4918 (2)	0.2472 (2)	0.07824 (9)	0.0695 (6)	
H4A	0.4477	0.3257	0.0733	0.083*	
H4B	0.5903	0.2531	0.0649	0.083*	
C5	0.27454 (16)	0.25192 (14)	0.20668 (7)	0.0377 (3)	
C6	0.30274 (16)	0.38724 (13)	0.29171 (7)	0.0370 (3)	
C7	0.36139 (17)	0.37550 (15)	0.34817 (7)	0.0416 (4)	
H7A	0.4372	0.3226	0.3545	0.050*	
C8	0.30729 (19)	0.44249 (15)	0.39539 (7)	0.0452 (4)	
H8A	0.3465	0.4350	0.4333	0.054*	
C9	0.19474 (19)	0.52025 (14)	0.38526 (7)	0.0450 (4)	
C10	0.13723 (18)	0.53487 (15)	0.32897 (8)	0.0459 (4)	
H10A	0.0626	0.5888	0.3226	0.055*	
C11	0.19215 (18)	0.46827 (14)	0.28228 (7)	0.0426 (4)	
H11A	0.1546	0.4779	0.2442	0.051*	
H1N	0.445 (2)	0.2955 (18)	0.2483 (9)	0.052 (5)*	

### Atomic displacement parameters (Å^2^)

**Table:**

	*U*^11^	*U*^22^	*U*^33^	*U*^12^	*U*^13^	*U*^23^
Cl1	0.0811 (4)	0.0609 (3)	0.0549 (3)	0.0125 (2)	0.0184 (2)	−0.0133 (2)
O1	0.0807 (11)	0.1309 (16)	0.0446 (7)	−0.0306 (11)	0.0110 (8)	−0.0221 (8)
O2	0.0341 (6)	0.0664 (8)	0.0511 (7)	−0.0016 (5)	0.0026 (5)	−0.0106 (5)
N1	0.0405 (7)	0.0582 (9)	0.0432 (7)	−0.0077 (6)	0.0057 (6)	−0.0150 (6)
N2	0.0347 (7)	0.0469 (8)	0.0404 (7)	0.0043 (6)	0.0000 (6)	−0.0087 (6)
C1	0.0687 (14)	0.115 (2)	0.0509 (12)	−0.0185 (14)	−0.0091 (11)	−0.0168 (12)
C2	0.0559 (11)	0.0712 (13)	0.0522 (10)	−0.0190 (9)	0.0065 (9)	−0.0229 (9)
C3	0.0364 (8)	0.0592 (10)	0.0475 (9)	0.0008 (7)	0.0050 (7)	−0.0114 (8)
C4	0.0668 (13)	0.0873 (16)	0.0543 (11)	−0.0210 (12)	0.0089 (11)	−0.0037 (10)
C5	0.0346 (8)	0.0410 (8)	0.0375 (8)	0.0013 (6)	0.0002 (6)	0.0008 (6)
C6	0.0376 (8)	0.0342 (8)	0.0391 (8)	−0.0014 (6)	0.0045 (6)	−0.0013 (6)
C7	0.0441 (9)	0.0378 (8)	0.0429 (9)	0.0044 (6)	−0.0011 (7)	−0.0006 (6)
C8	0.0537 (10)	0.0437 (9)	0.0383 (8)	−0.0004 (7)	0.0031 (7)	0.0000 (7)
C9	0.0537 (9)	0.0356 (8)	0.0457 (9)	−0.0019 (7)	0.0134 (8)	−0.0036 (6)
C10	0.0488 (9)	0.0354 (8)	0.0534 (10)	0.0062 (7)	0.0072 (8)	0.0019 (7)
C11	0.0475 (9)	0.0380 (8)	0.0422 (8)	0.0033 (7)	0.0007 (7)	0.0035 (6)

### Geometric parameters (Å, °)

**Table:**

Cl1—C9	1.7452 (16)	C3—C4	1.496 (3)
O1—C1	1.415 (3)	C3—H3A	0.9700
O1—C4	1.417 (3)	C3—H3B	0.9700
O2—C5	1.2390 (19)	C4—H4A	0.9700
N1—C5	1.351 (2)	C4—H4B	0.9700
N1—C3	1.453 (2)	C6—C7	1.385 (2)
N1—C2	1.457 (2)	C6—C11	1.386 (2)
N2—C5	1.373 (2)	C7—C8	1.389 (2)
N2—C6	1.4175 (19)	C7—H7A	0.9300
N2—H1N	0.84 (2)	C8—C9	1.379 (2)
C1—C2	1.499 (3)	C8—H8A	0.9300
C1—H1A	0.9700	C9—C10	1.381 (3)
C1—H1B	0.9700	C10—C11	1.381 (2)
C2—H2B	0.9700	C10—H10A	0.9300
C2—H2C	0.9700	C11—H11A	0.9300
			
C1—O1—C4	110.71 (16)	O1—C4—H4A	109.2
C5—N1—C3	126.33 (14)	C3—C4—H4A	109.2
C5—N1—C2	120.22 (14)	O1—C4—H4B	109.2
C3—N1—C2	113.14 (13)	C3—C4—H4B	109.2
C5—N2—C6	122.11 (14)	H4A—C4—H4B	107.9
C5—N2—H1N	117.2 (13)	O2—C5—N1	121.93 (14)
C6—N2—H1N	116.0 (13)	O2—C5—N2	122.28 (14)
O1—C1—C2	110.80 (19)	N1—C5—N2	115.78 (13)
O1—C1—H1A	109.5	C7—C6—C11	119.67 (15)
C2—C1—H1A	109.5	C7—C6—N2	119.12 (14)
O1—C1—H1B	109.5	C11—C6—N2	121.19 (14)
C2—C1—H1B	109.5	C6—C7—C8	120.16 (15)
H1A—C1—H1B	108.1	C6—C7—H7A	119.9
N1—C2—C1	109.42 (17)	C8—C7—H7A	119.9
N1—C2—H2B	109.8	C9—C8—C7	119.14 (16)
C1—C2—H2B	109.8	C9—C8—H8A	120.4
N1—C2—H2C	109.8	C7—C8—H8A	120.4
C1—C2—H2C	109.8	C8—C9—C10	121.34 (15)
H2B—C2—H2C	108.2	C8—C9—Cl1	119.59 (14)
N1—C3—C4	109.94 (15)	C10—C9—Cl1	119.06 (14)
N1—C3—H3A	109.7	C11—C10—C9	119.06 (16)
C4—C3—H3A	109.7	C11—C10—H10A	120.5
N1—C3—H3B	109.7	C9—C10—H10A	120.5
C4—C3—H3B	109.7	C10—C11—C6	120.59 (16)
H3A—C3—H3B	108.2	C10—C11—H11A	119.7
O1—C4—C3	112.21 (18)	C6—C11—H11A	119.7
			
C4—O1—C1—C2	−60.2 (3)	C6—N2—C5—N1	177.97 (15)
C5—N1—C2—C1	120.4 (2)	C5—N2—C6—C7	130.52 (17)
C3—N1—C2—C1	−53.6 (2)	C5—N2—C6—C11	−51.2 (2)
O1—C1—C2—N1	57.1 (3)	C11—C6—C7—C8	1.6 (2)
C5—N1—C3—C4	−121.82 (19)	N2—C6—C7—C8	179.86 (15)
C2—N1—C3—C4	51.7 (2)	C6—C7—C8—C9	0.1 (2)
C1—O1—C4—C3	58.8 (3)	C7—C8—C9—C10	−1.6 (3)
N1—C3—C4—O1	−53.6 (2)	C7—C8—C9—Cl1	179.34 (13)
C3—N1—C5—O2	166.36 (17)	C8—C9—C10—C11	1.3 (3)
C2—N1—C5—O2	−6.8 (3)	Cl1—C9—C10—C11	−179.61 (13)
C3—N1—C5—N2	−14.0 (2)	C9—C10—C11—C6	0.4 (3)
C2—N1—C5—N2	172.86 (16)	C7—C6—C11—C10	−1.8 (2)
C6—N2—C5—O2	−2.4 (2)	N2—C6—C11—C10	179.88 (15)

### Hydrogen-bond geometry (Å, °)

**Table:**

*D*—H···*A*	*D*—H	H···*A*	*D*···*A*	*D*—H···*A*
N2—H1N···O2^i^	0.838 (19)	2.114 (19)	2.9226 (19)	162.2 (19)

Symmetry codes: (i) *x*+1/2, *y*, −*z*+1/2.
